# Immobilization as a Strategy for Improving Enzyme Properties-Application to Oxidoreductases

**DOI:** 10.3390/molecules19078995

**Published:** 2014-06-27

**Authors:** Urszula Guzik, Katarzyna Hupert-Kocurek, Danuta Wojcieszyńska

**Affiliations:** University of Silesia in Katowice, Faculty of Biology and Environmental Protection, Department of Biochemistry, Jagiellonska 28, 40-032 Katowice, Poland; E-Mails: katarzyna.hupert-kocurek@us.edu.pl (K.H.-K.), danuta.wojcieszynska@us.edu.pl (D.W.)

**Keywords:** oxidoreductases, immobilization, stability of enzyme

## Abstract

The main objective of the immobilization of enzymes is to enhance the economics of biocatalytic processes. Immobilization allows one to re-use the enzyme for an extended period of time and enables easier separation of the catalyst from the product. Additionally, immobilization improves many properties of enzymes such as performance in organic solvents, pH tolerance, heat stability or the functional stability. Increasing the structural rigidity of the protein and stabilization of multimeric enzymes which prevents dissociation-related inactivation. In the last decade, several papers about immobilization methods have been published. In our work, we present a relation between the influence of immobilization on the improvement of the properties of selected oxidoreductases and their commercial value. We also present our view on the role that different immobilization methods play in the reduction of enzyme inhibition during biotechnological processes.

## 1. Introduction

The immobilization of enzymes on different carriers is an important challenge in biotechnology. Hitherto a great variety of designed systems have been examined and discussed. The main aim of immobilization is to obtain stable and reusable enzymes with resistance to different environmental factors [1,2]. Among the various types of enzymes, in particular multimeric enzymes, such as oxidoreductases, are not stable under various conditions. It was shown that hydrostatic and osmotic pressure, temperature or pH might cause subunit dissociation. However, stabilization of multimeric enzymes may be achieved by medium engineering, chemical crosslinking, protein engineering or enzyme immobilization [3,4,5]. In the 1970s Katchalski *et al.* [6] demonstrated that enzymes immobilized on surfaces and confined in cavities exhibit higher activity than in free solution. On the other hand, pH, immobilization time and protocol, surface coverage, and surface curvatures and chemistries can substantially impact the enzymatic activity of multimeric proteins [7]. Stabilization of multimeric enzymes was observed after immobilization by entrapment, covalent immobilization and physical adsorption. This effect is connected with interaction between the support and the enzyme. Covalent immobilization and physical adsorption lead to binding maximal amount of enzyme subunits with maximal area of support. After the enzyme entrapment, hydrophobic or ionic interactions decrease not only dissociation of protein into subunits but also formation of an inactive intermolecular aggregate [4]. 

Immobilization improves control of the reaction by the possible use of different reactor configurations. Furthermore, the immobilized enzymes exhibit higher selectiveness and specificity [8]. Nevertheless, during the immobilization procedure enzymes may be denatured and lose their activity. It is caused by distortions, especially if some multi-interactions between the enzyme and matrix occur. It has been reported that the immobilization of proteins on solid substrates may cause secondary structural changes [9]. These changes lead to proteins losing their α-helical structure and gaining β-sheet structure [10,11]. On the other hand, the distortions may be responsible for altered properties of the biocatalyst [12]. 

In recent years, various oxidoreductases especially laccases, tyrosinases, peroxidases and dioxygenases have found applications in many technological processes [13,14,15]. It was demonstrated that the immobilization of dioxygenases, which is characterized by low operational stability, allows for an increase in its biotechnological value [16,17,18]. In turn, polyphenol oxidases and peroxidases are enzymes which most often require mediators for their activities. Therefore, the immobilization of such enzymes on insoluble carriers facilitates construction of stable systems of the electron chain [19]. Because of an increasing interest in the biotechnological use of oxidoreductases it seems very important and necessary to present the actual knowledge about the relationship between the immobilization and application value of these enzymes. 

## 2. Immobilized Polyphenol Oxidases–Valuable Enzymes in Biotechnology

Polyphenol oxidases are copper containing oxidoreductases that catalyze transformation of a large number of phenolic and non-phenolic compounds. They are subdivided into two groups: laccases and tyrosinases [14,20,21]. 

**Figure 1:**
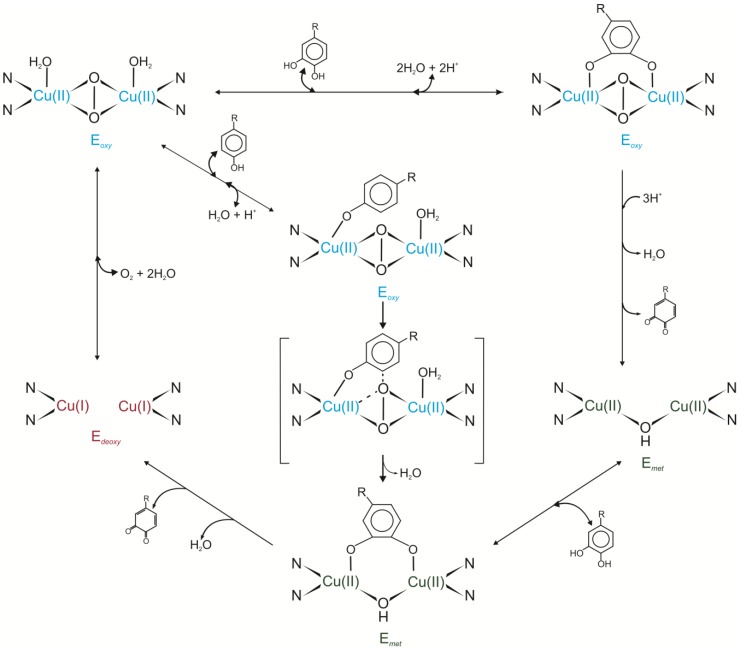
Catalytic cycles of tyrosinase [14,22,23].

Tyrosinases play a crucial role in the hydroxylation of monophenols to *ortho*-diphenols [E.C.1.14.18.1] and oxidation of *ortho*-diphenols to *ortho*-quinones [E.C.1.10.3.1]. In the catalytic centre of these enzymes two copper ions—CuA and CuB—are located. The oxidation state of the copper and absence/presence of the oxygen in the active site of tyrosinases determine the kind of the enzyme form called *met*, *oxy* and *deoxy*. The binding of the oxygen to the *deoxy*-form of tyrosinase initiates enzyme activity by formation of the *oxy*-form of the enzyme. This form may interact with monohydroxylated or dihydroxylated substrate. In the first case, dioxygen attacks the phenolate ring which leads to O-O bond cleavage and results in the hydroxylation of the substrate to *o*-diphenol and subsequent oxidation of *o*-diphenol to *o*-quinone. In the second case, a dihydroxylated substrate coordinates two Cu ions. Released quinone leads to the formation of the *met*-form of the enzyme to which only dihydroxylated substrate may bind. After this reaction both Cu ions are coordinated by the oxygen from the substrate. Dissociation of the quinone product from the catalytic site of the enzyme results in the regeneration of the *deoxy*-form of tyrosinase (Figure 1) [14,22,23]. 

Laccases [E.C.1.10.3.2.] are cuproproteins belonging to the class of oxidoreductases and catalyze one-electron oxidation of the substrate by generating a free radical. The product of this reaction is unstable and may undergo a second, enzymatic or non-enzymatic (hydratation, disproportionation, polymerization) reaction. The active site of laccases consists of four copper atoms classified into three different types according to their paramagnetic resonance spectrum [14,21,24]. Laccases oxidize a substrate by the four-electron reduction of dioxygen to water. Substrate oxidation takes place at the type 1 copper (T1) whereas T2/T3 triatomic copper cluster is responsible for oxygen reduction. T1 copper accepts an electron from the electron-donating substrate. This electron is transferred to the T2/T3 copper cluster. 

**Figure 2:**
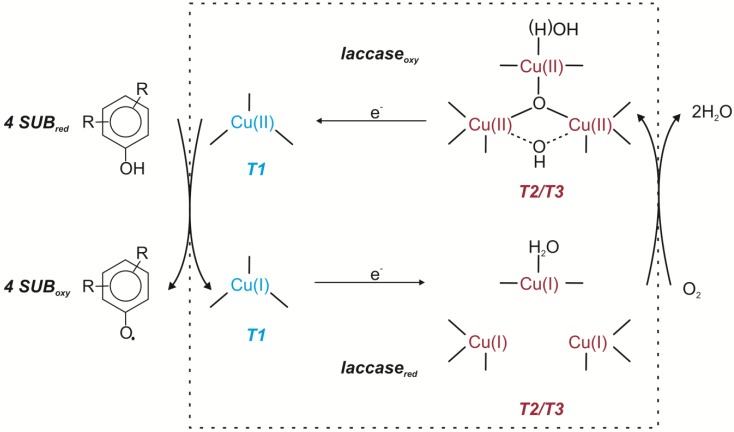
Catalytic cycles of laccase [14,19,25,26].

After interaction between the oxygen and T2/T3 cluster two-electron reduction occurs. It leads to the formation of the peroxide intermediate containing the dioxygen anion. The peroxide intermediate undergoes two-electron reduction with subsequent O-O bond cleavage. As a consequence a native intermediate is produced. This intermediate is a fully oxidized form connected with the product of full dioxygen reduction. Slow decay of the native intermediate leads to generation of the resting fully oxidized form (Figure 2) [14,18,24,25,26]. 

In recent years, increasing interest has been directed at the use of polyphenol oxidases for detoxification of environmental pollutants, organic synthesis, construction of biosensors and bioactive compounds, for their application in pulp and paper, as well as dye, textile and cosmetic industries [27,28]. However, a number of practical problems such as the high cost of enzyme isolation and purification, non-reusability, the instability of the protein structure or sensitivity to the harsh process, conditions make the full usage of these oxidases difficult. Many of the above limitations may be removed by the immobilization of enzymes [24,26].

### 2.1. Application of Immobilized Tyrosinase in Biotechnology

Recently, the applications of electrochemical biosensors has increased because, in comparison with traditional analytical methods, they provide faster detection and are more sensitive and selective. Biosensors based on the immobilization of tyrosinase are an alternative to other techniques as they combine biological recognition through enzyme specificity with construction simplicity [29,30]. A variety of matrices have been extensively used to immobilize tyrosinase, including carbon paste, conducting polymer- modified electrodes, silica sol-gel composite films, Langmuir-Blodgett thin films, and layer-by-layer films [29]. 

Carbon nanotubes are a man-made form of carbon which characterizes unique chemical, physical, optical and magnetic properties. They are used in a large variety of application such as devices in nanoelectronics. Developing nanocomposites with carbon nanotubes also improves their function as structural element for the immobilization of enzymes during the construction of biosensors [31,32]. Carbon nanotubes offer a high surface area for enzyme loading, with the establishment of hydrophobic or electrostatic interaction for the better binding between the enzyme and the support, as well as biocompatibility and mechanical resistance [33]. In order to be used in biosensors they have to be modified. Because vinyl monomers possess hydrophobic vinyl groups and hydrophilic functional groups, various vinyl monomers were grafted into the multi-walled carbon nanotube (MWCNT). The multi-walled carbon nanotubes were modified by radiation-induced graft polymerization at room temperature and in aqua solution. The ionic property-modified multi-walled carbon nanotubes constituted better support for the immobilized tyrosinase because of strong cationic property. The ionic property-modified MWCNT electrode was constructed by Ryu and Choi [32] for determination of caffeine. This biosensor was more sensitive for this compound than the free enzyme in solution, because of the increased access of substrate molecules to the enzyme catalytic site [32].

A mediator-free tyrosinase biosensor was constructed for the determination of phenolic compounds. It was shown that the nano-ZnO matrix with a high isoelectronic point (pI 9.5) provided a microenvironment of a favorable isoelectronic point for tyrosinase loading (pI 4.5). Moreover, ZnO also promoted the electron transfer between the tyrosinase and the electrode to a large extent [34]. In 2006 Li *et al.* [35] constructed a glass carbon/ZnO/chitosan/tyrosinase electrode. They showed that the ZnO nanoparticles were regularly dispersed in chitosan solution exhibiting uniform porous structure which provides a significant increase of the effective surface for tyrosinase loading. Moreover, this structure enables the specific reaction between the substrate and the enzyme. Therefore, the described biosensor was more sensitive for the substrates and respond faster than the free enzyme. Additionally, the biosensor activity decreased gradually. After 20 days this electrode retained 91% of the initial current response [35]. 

ZnO was also used during fabrication of Zinc Oxide-Nafion (silica-perfluorosulfonated ionomer) and multi-walled carbon nanotube-Zinc Oxide-Nafion (MWCNT-ZnO-Nafion) electrodes. Comparison of the results obtained for both electrodes showed that after using of MWCNT-ZnO-Nafion, the sensitivity and stability of tyrosinase biosensor increased. The shorter response time of this biosensor may be mainly ascribed to the relatively large pore size. Moreover, biosensor based on the MWCNT-ZnO-Nafion characterizes better long-term storage stability and a lower detection limit (30 nM) than that obtained for the biosensor based on ZnO-Nafion composite without MWCNT (58 nM). However, immobilization on MWCNT-ZnO-Nafion did not affect stability of the enzyme at a different pH. It indicates that the immobilization of tyrosinase in the MWCNT-ZnO-Nafion composite film does not change the microenvironment of the enzyme. The smaller K_m_ values indicate that the affinity of the enzyme immobilized in MWCNT-ZnO-Nafion matrix to phenol and its derivatives is stronger than in other matrices because of good mass transport within the composite film [34]. 

Carbon nanotubes as an activating tyrosinase support were used not only to prepare phenol assaying electrode but also for the selective synthesis of phenol derivatives such as different catechols [33]. The immobilization of tyrosinase on the multiwalled carbon nanotubes by the layer-by-layer procedure was used to selectively synthesize catechols from *para-* and *meta*-substituted phenols in the organic solvent. During preparation, MWCNTs were treated with poly(diallyldimethylammonium chloride) (PDDA) and then the enzyme, negatively charged at the operative pH, was applied on PDDA as a polycation. It is known that the use of enzymes in organic solvents enhances solubility of hydrophobic substrates, reduces side reactions and enhances catalyst performances. Results obtained by Subrizi *et al.* [33] showed that the immobilization of tyrosinase on the described support caused higher stability of the enzyme under hydrophobic condition than in the buffer. Additionally, kinetic parameters indicated that the immobilized enzyme showed higher rates of reaction than the native one and, what is more interesting, immobilized tyrosinase was able to catalyze oxidation of phenol derivatives which were not transformed by the native enzyme [33].

Apetrei and Apetrei *et al.* [29,30] investigated tyrosinase biosensors for the detection of biogenic amines after the immobilization of the enzyme on/in different carriers. The usage of tyrosinase immobilized on the single-walled carbon nanotube-modified glassy carbon electrode (SWCNT-GCE) for the detection of epinephrine caused a faster response rate than that reported for the silica sol-gel matrix. It was connected with rapid electron transfer between the enzymatically produced epinephrine-quinone and the electrode. SWCNTs as a carrier are attractive because of a lager surface area for the enzyme interaction than the multi-walled carbon nanotubes (MWCNTs). The values of catalytic parameters obtained for the biosensor indicated that SWCNT nanocomposite film creates a proper environment for enzyme immobilization. The immobilized enzyme retains its properties even in the case in which it is one molecule the movement of which is completely limited or restricted to a small region. Moreover, this matrix may connect electrodes with various kinds of biomolecules [29]. High sensitivity and a fast response rate were also observed for the amperometric detection of tyramine and dopamine after cross-linking the immobilization of tyrosinase into polypyrrole [30].

Tyrosinases catalyze the conversion of phenolic substrates to the corresponding quinones which may interact with a color reagent, 3-methyl-2-benzothiazolinone (MBTH). The resultant red product of this reaction may be spectrophotometrically detected. For that reason immobilized tyrosinase in the form of an optical biosensor can be used as an alternative to the conventional methods of detecting chemical compounds. The preponderance of optical biosensors results from their particularly high sensitivity, small size and cost effectiveness [13,36,37]. Tyrosinase immobilized on jellose/agarose membrane was used to detect and estimate micromolar quantities of 3,4-dihydroxyphenylalanine (L-DOPA)—a clinical indicator in the detection of pigmentory disorders. However, this matrix is semi-opaque and the absorption of the light passing through the matrix causes only partial work of the sensor [36]. For this reason chitosan seems to be a better matrix for the construction of the optical biosensors. Chitosan surface is transparent in the UV and visible spectrum and should not have a significant negative influence on optical detection methods. Abdullah *et al.* [13] constructed a sensor based on tyrosinase immobilized on chitosan, which was stable for at least two months and characterized limit detection 0.9, 1.0, 1.0 and 3.0 µM for 4-chlorophenol, phenol, *m*-cresol and *p*-cresol, respectively. A proportional increase of color intensity with increasing the substrate concentration was observed after 15 minutes of the sensor’s exposure to the substrate [13]. A remarkably shorter time (5 min) of linear response of optical biosensor for the presence of phenolic pollutants in water was obtained by Russel and Burton [37] after the immobilization of tyrosinase on the nylon membrane. These kinds of optical biosensors are portable and relatively cheap, therefore they could be used by non-experts to detect various phenols.

Phenolic compounds are some of the more important environmental pollutants generated by paint, pesticide, coal conversion, polymeric resin, petroleum and petrochemicals industries [38,39]. They can be removed from the environment with the use of various oxidoreductive enzymes. Tyrosinase-mediated dephenolization outshines processes catalyzed by other enzymes, because tyrosinase, apart from molecular oxygen as an oxidant, does not require stoichiometric quantities of other reagents [38,40]. Xu and Yang [39] demonstrated the use of immobilized tyrosinase for the treatment of phenol-polluted water resulting in decreased toxicity of phenolic compounds in this environment. Their experiment showed that while both phenols mixture and *p*-chlorophenol were highly harmful to *Hydra sinensis*, toxicity of these compounds was efficiently diminished by treatment with immobilized tyrosinase [39].

Although soluble tyrosinase may be used for dephenolization, immobilization increases enzyme stability and effectiveness [38,40]. The thermal stability of an immobilized enzyme is one of the most important criteria of their application. Bounding of the enzyme to the matrix makes it more resistant against denaturing agents and heat. Therefore, the immobilized enzyme has a higher thermal stability, which is connected with the reduction of its conformational flexibility. Dincer *et al.* [38] showed that at 50 °C free tyrosinase lost 86% of activity, whereas an immobilized enzyme only 48%. Additionally, immobilized tyrosinase had no significant activity losses within 30 days when stored at −18 °C. It is proposed that the immobilization of tyrosinase on chitosan-clay composite crosslinked with glutaraldehyde preserves a tertiary structure of the protein [38]. A similar effect was obtained after the immobilization of tyrosinase on diatom biosilica modified with 3-aminopropyl triethoxysilane (APTES) and non-modified glutaraldehyde diatom biosilica. The reduction of conformational changes of the immobilized enzyme caused higher thermal stability of the immobilized tyrosinase in comparison to the free enzyme. At 70 °C, after one hour of incubation, immobilized tyrosinase retained 9% of its initial activity, whereas the free enzyme lost all its initial activity after 30 min of incubation [40]. Phenol and its derivatives at certain concentration, similar to extremely high and low pH, may cause denaturation of enzymes or weakened subunit-subunit interaction. For that reason multipoint covalent attachment of multi-subunit enzymes with the matrix has a significant influence on their usage in the presence of denaturing agents [39,40]. The immobilization of tyrosinase in the form of cross-linked enzyme aggregates (CLEAs) crosslinked with glutaraldehyde allows for phenol removal over a broad range of pH (5.5–8.5). This kind of immobilization enabled the complete conversion of phenol, *p*-cresol, *p*-chlorophenol and bisphenol A within 0.5–3.0 h, depending on the enzyme dosage and the type and initial concentration of the target phenol. However, because tyrosinase CLEAs have a few disadvantages such as inducing flocculation of particles which are not dispersed well in the solution, their practical applications are limited. Therefore, the combination of CLEA technology with traditional immobilization methods, for example entrapment of the CLEAs into calcium alginate, seems to solve this problem [39]. Xu and Yang [39] demonstrated that tyrosinase immobilized in this way can be applied in continuous stirred tank reactor. A continuous dephenolization process took place for over 26 h. Phenol concentration decreased from 2.5 mM to 1.5 mM during the first 5 h and then reached a constant level for the later period. Tyrosinase CLEAs entrapment into calcium alginate gel has possible applications in wastewater treatment [39]. 

The mmobilization of tyrosinase may be carried out by different methods and with a large number of supports. It increases application properties of this enzyme, especially in bioremediation processes and in electrobiochemistry.

### 2.2. Useful Laccase Properties Obtained after Immobilization

Laccases are ubiquitous enzymes present in higher plants, bacteria, fungi, insects and lichens. Due to their high efficiency they have found applications in bioremediation, chemical synthesis, biosensing, for biobleaching of paper pulp or fabric finishing. However, difficulties in the separation of laccases from the reaction systems limit industrial application of these enzymes. Therefore, their immobilization improves the enzymes’ recoverability together with activity, storage, and operational stability [41,42]. 

Due to high efficiency and low-cost degradation of the pollutant, laccases are frequently used for the treatment of contaminated environments [43]. For example, Liu *et al.* [43] examined laccase immobilized on carbon-based mesoporous magnetic composites (CMMC) in phenol and *p*-chlorophenol removal. Combining the magnetic bio-separation technology with the immobilized enzyme enabled easier separation and recovery of the catalyst and a reduction of costs [41,43]. It was also shown that laccase immobilized on CMMC had a significantly broader pH and temperature profile than the free enzyme. The same effect was observed for laccase immobilized on polyacrylonitrile (PAN), the widely studied support showing good mechanical properties, solvent and abrasion resistance and high tensile strength [44,45]. The kinetic parameters of reactions catalyzed by laccase immobilized on CMMC indicated less affinity for the substrate than that of the free enzyme. Nevertheless, the immobilized enzyme during 12 h utilized 78% and 84% of phenol and *p*-chlorophenol, respectively [43]. A similar high efficiency of degradation was observed by Wang *et al.* [46] and Xu *et al.* [47]. Laccase immobilized on magnetic Cu^2+^-chelated silica support removed pentachlorophenol with 82.9% efficiency [46], whereas conjugation of the enzyme onto the surface of polyacrylonitrile electrospun fibrous membrane resulted in 87% efficiency of 2,4,6-trichlorophenol removal in four hours [47]. 

The operating stability of the immobilized enzyme is a very important parameter. Immobilized enzymes may be easily separated from the reaction solution and reused, which greatly decreases costs of the enzyme and increases its significance for practical application [43,45,47]. The immobilization of laccase from *Trametes versicolor* on the controlled porosity carrier (CPC) silica beads allowed laccase to remain stable and maintain more than 85% of its initial activity after 30 days. After immobilization, laccase degraded more than 90% of 2,4-dinitrophenol within 12 h of treatment [41]. In turn, the use of laccase immobilized on polyacrylonitrile beads in the fluidized bed reactor obtained almost 100% removal efficiency of bisphenol A (4,4'-isopropylidenediphenol), tetrachlorobisphenol A (2,2-bis-(3,5-dichloro-4-hydroxyphenyl), bisphenol B (2,2-bis-(4-hydroxyphenyl)butane) and bisphenol F (bis(4-hydroxyphenyl)methane)—compounds causing adverse alterations in reproductive and development processes, as well as metabolic disorders. The immobilized enzyme retained over 85% of its original activity after 30 days [44]. Decrease in the activity of the immobilized laccase as a result of repeated usage may be expected due to the possibility of enzyme denaturation during the operation process. What is interesting, Wang *et al.* [45] observed the highest activity of commercial laccase immobilized on polyacrylonitrile or polyacrylonitrile/montmorillonite/graphene oxide (PAN/MMT/GO) at the second or third cycle. It is possible that during repeated usage, the membrane became slack and downy, contributing to more sites for the enzyme to reach the substrate [45].

Reactive dyes are extensively used in the textile industry mainly due to their effective binding to the textile fibers. Most of them are stable and nondegradable by conventional methods and their presence in the environment poses a health hazard. Additionally, the presence of color in water may affect the transmission of light and photosynthesis, and reduce aquatic diversity. Therefore, decolorization of waste waters discharged in textile processing is a major problem. In recent years it was shown that many industrial dyes could be decolorized by fungal laccases frequently immobilized on different supports, such as glass-ceramic materials, imidazole-modified silica, montmorillonite, alginate-gelatin mixed gel, hydrophobic sol-gel and green coconut fiber [27,48,49,50,51,52]. 

Laccases physically entrapped or immobilized by their covalent attachment to various supports are the most commonly used in decolorization processes [48,49,50,52]. The decolorization of waste waters by immobilized laccases is the result of enzymatic catalysis and support adsorption [49,50,52]. It was shown that alginate and chitosan are biopolymers, which can be used as sorbents to remove dyes from the aquatic solutions. Although the entrapment of laccases in alginate mixed gels or hydrophobic Sol-gel decreases their activity and dye affinity, it improves their pH stability, thermostability and enables reusability of the enzymes [50,51]. Additionally, the immobilization of laccases on such insoluble supports limits their conformational changes that increases their operational stability and durability [50,52]. For example, the entrapment of laccase in alginate-gelatin, alginate-chitosan mixed gels or in hydrophobic Sol-gel matrix of trimethoxysilane and proplytetramethoxysilane led to its significant stability towards heat denaturation. However, under these conditions lower affinity of the enzyme to the substrates caused by diffusional limitations and decreased protein flexibility was observed [48,50,51]. 

In turn, Cristόvoã *et al.* [49] immobilized a commercial laccase on green coconut fibers and verified the ability of the enzyme to decolorize different textile dyes. They employed two different strategies of enzyme immobilization: one-point (pH 7.0) and multipoint (pH 10.0) covalent attachment. The covalent binding provides strong and stable enzyme attachment, avoiding the enzyme desorption or conformational changes when exposed to some medium variations, and makes the laccase more attractive for industrial applications. It was shown that the commercial laccase immobilized by one-point covalent attachment obtained higher activity and affinity to the dyes, whereas immobilization at pH 10.0 improved the biocatalyst thermal stability at 50 °C. It was suggested that the additional bonds established between the enzyme and support during immobilization by multipoint covalent attachment promoted the stabilization of the immobilized enzyme [49]. Decolorization is limited by the concentration of a mediator such as 1-hydroxybenzitriazole (HOBT) and 2,2'-azinobis(3-ethylbenzthiazoline-6-sulfonate) (ABTS) [48,49,52]. The addition of ABTS during dye decolorization by laccase immobilized on green coconut fiber significantly increased enzyme activity [49]. The same effect was observed during decolorization carried out by laccase entrapped in the hydrophobic Sol-gel [48].

The desired properties of immobilized laccases such as improved characteristics, stability and reusability obtained after they are immobilized on different carriers show the suitability of these biocatalysts for continuous treatment of different industrial effluents.

## 3. Immobilized Peroxidases as Useful Tools in Biotechnology

**Figure 3:**
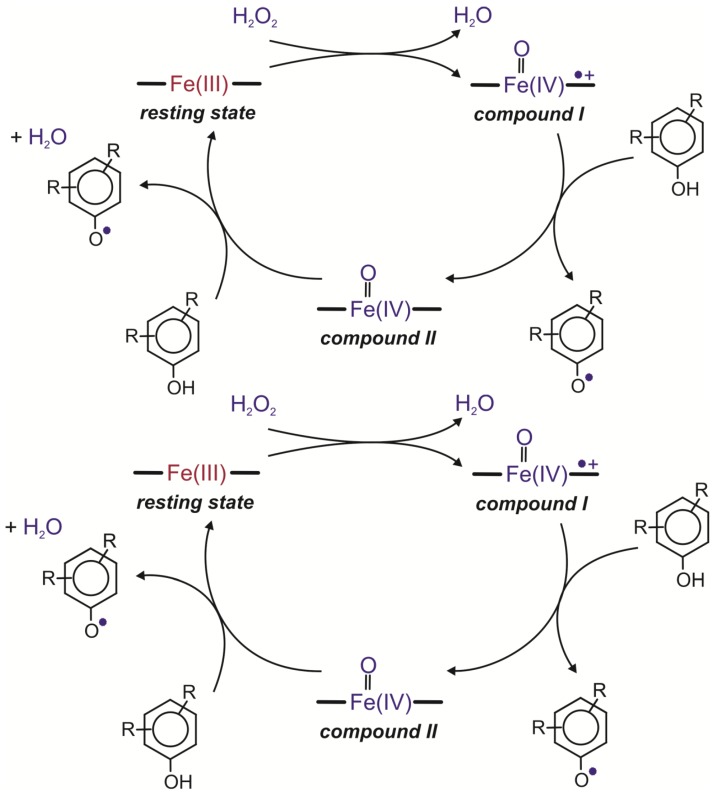
Catalytic cycles of peroxidases [15,58,59].

Peroxidases [E.C.1.11.1.7] belong to oxidoreductases which are found in animals, plants and microorganisms. They oxidize many aromatic compounds in the presence of hydrogen peroxide and form polymeric products [53,54,55,56]. The catalytic mechanism of horseradish peroxidase is the best described among the proteins of this class, and it is considered as a model system of peroxidase reaction [57]. The first step in the catalytic cycle of this enzyme is interaction between the Fe(III) resting state of the enzyme and hydrogen peroxide. During this reaction, a water molecule is released with the simultaneous two-electron oxidation of the heme iron to compound I, a high oxidation state intermediate comprising an Fe(IV) oxoferryl centre and a porphyrin-based cation radical (Por**^·^**^+^Fe^IV^=O). The second step comprises two single-electron transfers. It leads to the conversion of compound I to the resting state of the enzyme. The first one-electron reduction of the porphyrin radical cation requires the presence of a reducing substrate and leads to the generation of compound II, an Fe(IV) oxoferryl state (Fe(IV)=O). The second one-electron reduction step returns compound II to the resting state of the enzyme (Figure 3) [15,58,59].

Peroxidases have found applications in different industrial processes due to their high stability in the solution, broad substrate specificity and tolerance to the wide range of pH and temperature, and easy availability from plant materials [55,60]. 

Like laccases, peroxidases are frequently used in the decolorization of textile wastewater effluents, and many different techniques were reported for enhancing their activity and stability. Most often physical entrapment of these enzymes in various gel carriers or their immobilization by covalent attachment was used [54,55,61,62,63]. The entrapment of peroxidases into a sol-gel matrix of tetramethoxysilane and proplytrimethoxysilane or in alginate gel and mixed alginate-pectin gel improves its storage stability, pH stability and thermostability. It also increases enzymes reusability and efficiency of decolorization [55,62,63]. For example, the Sol-gel matrix encapsulated manganese peroxidase from *Ganoderma lucidum* showed 84.6% of its initial activity after 10 periodic oxidation cycles and decolorized Crescent (99.2%), Magna (94.6%), Arzoo (89.6%) and Chenab (78.5%) textile effluents. An increase of the decolorization process was correlated with an increased reaction time [62]. 

Jamal *et al.* [63] prepared a peroxidase-concanavalin A complex and immobilized it on calcium alginate pectin gel. The obtained complex demonstrated increased storage stability in comparison with the free peroxidase and non-immobilized complex of peroxidase-concanavalin A. The entrapped complex retained 59.6% of the initial activity after 50 days of incubation. Moreover, the immobilized complex decolorized Disperse Red 19 and Disperse Red 19 with Disperse black 9 mixtures (91.2% and 82.1%, respectively). 

Lignocellulosic fibers, such as corncobs possess hydroxyl groups which may react with other polar functional groups. These reactive hydroxyl groups create a chemical linkages for the immobilization of enzymes [54,64]. Galárraga *at al.* [54] performed multipoint covalent immobilization of peroxidase on corncob powder-glyoxyl support at pH 10.0 using the three-dimensional aminated surface area of the enzyme. At this pH, deprotonated amine groups enabled a nucleophilic attack of the support on the aldehydes that led to the formation of the corncob powder-glyoxyl aminated peroxide derivatives. Multipoint covalent bonds increased the rigidity of peroxidases and limited the conformational changes of the enzymes which caused greater resistance to temperature, organic solvents, denaturing agents and other factors. Peroxidase from soybean coat immobilized by covalent attachment on highly activated corncob powder was employed for decolorization of 0.02 mM bromophenol blue. It was shown that aminated peroxidase decolorized more than 90% of this dye. Moreover, the immobilized enzyme retained 60% of the activity after three catalytic cycles [54]. 

Horseradish peroxidase (HRP) has been successfully used for the elimination of phenol from aqueous solution [61]. Due to the high costs of the enzyme, reactors containing an immobilized enzyme may be used for the treatment of large volumes of waste waters [61]. Pramparo *et al.* [65] used covalent immobilization of the horseradish peroxidase on Eupergit^®^C epoxy-activated support, which is resistant to mechanical and chemical stresses, and adaptable to a variety of configurations and specific processes, carried out in reactors. In this work, three different procedures of HRP immobilization were described: direct binding of HRP to the Eupergit^®^C *via* its oxirane groups, HRP binding to the beads *via* a spacer made from adipic dihydrazine (hydrazido Eupergit^®^C) and HRP binding to hydrazido Eupergit^®^C through the enzyme carbohydrate moiety previously modified by periodate oxidation. The immobilized HRP demonstrated the same activity after using different methods of immobilization. However, the third procedure of immobilization obtained the same enzyme activity, but with a reduced amount of the enzyme. The elimination of phenol in the stirred reactor reached 92% with the use of 0.006 U/mL of the immobilized HRP, whereas the same degradation rate was obtained after using 1.000 U/mL of the free HRP [65]. Slightly higher efficiency of phenol removal (93%) was obtained using HRP immobilized on aminopropyl inorganic porous Celite beads in a membraneless electrochemical reactor [66]. Unfortunately, practical application of peroxidases in degradation of aromatic compounds has some limitations, mainly due to the need for a continuous external supply of hydrogen peroxide. The solution to this problem is the application of electroenzymatic process which allows combining enzymatic oxidation with electrogeneration of hydrogen peroxide. Cho *et al.* [66] demonstrated that immobilized HRP in a membraneless electrochemical reactor effectively degrades phenol into *p*-benzoquinone, organic acids such as maleic acid, oxalic acid and malonic acid, and carbon dioxide. 

From the economical point of view, the usage of an immobilized enzyme is cost-effective when the enzyme reaction is continuous. One of the biggest problems in continuous processes is operational stability of the enzyme. Bayramoğlu and Arıca [53] examined the operational stability of HRP immobilized on magnetic poly(glycidylmethacrylate-co-methylmethacrylate) (poly(GMA-MMA)-GA) beads in magnetically stabilized fluidized bed reactor at 25 °C for 48 h. This reactor was continuously supplied with 1 mM phenol and/or *p*-chlorophenol. After 48 h of continuous work of the reactor, the enzyme retained about 92% and 79% of its initial activity with phenol and *p*-chlorophenol, respectively. The higher operational inactivation rate of HRP during *p*-chlorophenol oxidation was probably connected with releasing chlorine ions during the enzymatic process and enzyme inactivation [53]. 

Promising results during *p*-chlorophenol degradation by soybean peroxidase (SBP) and horseradish peroxidase immobilized on porous aldehyde glass in stirred batch reactor were obtained by Bódalo *et al.* [67]. The conversion efficiency of 2 mM *p*-chlorophenol with immobilized SBP amounted to 95% after 30 min. Immobilized HRP needed 60 min to reach 90% conversion of this substrate concentration. Although hydrogen peroxide is a co-substrate in the reaction catalyzed by peroxidases, its higher concentrations may inactivate these enzymes. Bódalo *et al.* demonstrated that immobilized SBP was less susceptible to inactivation by hydrogen peroxide than immobilized HRP and free SBP. Similar results were obtained by Gómez *et al.* [61] for peroxidases immobilized on glutaraldehyde-activated aminopropyl glass beads. These results are remarkable and indicate that the use of the immobilized SBP is a cheaper alternative to HRP for *p*-chlorophenol removal [60,61,67]. 

A large surface-to-volume ratio of nanometer scale materials in comparison with traditional macroscale materials makes them a promising support for enzyme immobilization. Reduction of the dimension of the enzyme carrier materials improves the catalytic efficiency of the immobilized enzyme because of reduction of diffusion limitation [68,69]. Due to the hollow and porous structure and large surface area, nanotubes seem to be the most efficient support for the immobilization of peroxidases. Chitosan-halloysite hybrid-nanotubes were used for the horseradish peroxidase immobilization. Halloysite nanotubes are two-layered aluminosilicate with negative potential at pH 6.0–7.0, but theirs inorganic properties and unfavorable biocompatibility result in the weak binding of the enzyme to the support, and low enzyme loading. These disadvantages may be overcome by halloysite nanotubes modification with chitosan and glutaraldehyde. This kind of nanocomposite provides more functional groups such as amino, aldehyde, hydroxyl or carbonyl for enzyme binding and allows in this way higher enzyme loading. The amount of protein yield after the immobilization of HRP on chitosan-halloysite hybrid-nanotubes was 21.5 mg HRP/g support [69]. Such immobilization efficiency was comparably higher than for supports previously reported for HRP such as 3.35 mg/g on magnetic poly(GMA-MMA)-GA beads or 3.7 mg/g on aminopropyl inorganic porous Celite beads [53,66]. The allophone-nanoclay, natural inorganic soil fraction characterized by a high superficial area and structural stability, may be the alternative for inorganic nanomaterials. Allophone was examined as a support for the immobilization of manganese peroxidase (MnP) from *Anthracophyllum discolor*, an enzyme useful in the degradation of polycyclic aromatic hydrocarbons (PAHs). Nanoclay-immobilized MnP, in comparison to the free enzyme, showed better stability at a higher temperature and a broader pH range [67]. Alteration of immobilized enzyme activity *versus* pH profile indicated that the immobilization of enzymes on surface-charged materials may lead to the displacement of the pH-activity profile to acidic or alkaline regions [68,70]. The shift of the optimal pH of the immobilized enzyme activity may results from pH dependent modification of peroxidase structure or the fact that the pH in the region of the active site of the immobilized enzyme is lower than the pH in the solution bulk. The storage time of immobilized MnP was also significantly higher. After 6 months of storage at 4 °C only about a 20% loss of immobilized enzyme activity was observed, whereas free MnP under the same conditions lost about 87% of its initial activity. Moreover, MnP immobilized on nanoclays showed enhanced anthracene transformation in soil. It suggests that allophone-immobilized MnP may be an efficient alternative for *in situ* bioremediation on a large scale [68].

Peroxidases have also found application in the construction of biosensors, especially those that are used for determining hydrogen peroxide, phenolic compounds, metal ions or the total level of biogenic amines [71,72,73,74,75,76,77,78]. In order to improve the analytical possibilities of electrochemical biosensors constructed on the basis of immobilized peroxidases, studies on the improvement of immobilization methods, on miniaturization of the sensor elements and on increasing the enzyme stability are conducted [76].

In recent years, methods have been developed of trace metal ions measurement based on the inhibitory effect of metals on peroxidase activity. The peroxidase-based electrochemical biosensors often contain enzymes immobilized on multiwalled carbon nanotubes [74,75]. The improvement of analytical characteristic of such biosensors was obtained by maize tassel (MT) dispersing on the nanotubes. Current response of the peroxidase/maize tassel-MWCNT depends on the ratio MT:MWCNT and reaches its maximum at 4:1. The second factor which influences analytical sensitivity of these kinds of biosensors is enzyme loading. The response of the peroxidase/MT-MWCNT biosensor for copper(II) ions detection increased up to a maximum of 10 mg/mL. For higher concentration (above 10 mg/mL) the sensitivity of the biosensor decreased, probably due to diffusion limitation. Concentration of nafion, which binds peroxidase to the matrix, affected the electrocatalytic response of this biosensor. It was found that the response of peroxidase/MT-MWCNT biosensor increased slightly at the concentration of nafion from 0.1 up to 0.3% and decreased at a higher concentration of this compound. The peroxidase/MT-MWCNT biosensor showed sensitivity in the range of 0.068–2.0 mg/L Cu^2+^ with a detection limit of 4.2 µg/L [75]. A similar biosensor was applied for the detection of Zn^2+^ in aqueous solution, in the range of 0.35–12 mg/L with a detection limit 7.5 µg/L [69]. Since the construction of the described biosensors do not require any complicated immobilization procedures, and due to their repeatability, reproducibility and high selectivity, they can be used as a management tool for determining the trace metal ions [74,75]. 

Single walled carbon nanotubes (SWNTs) form the basis of hydrogen peroxide biosensors. Large length-to-diameter and good conductivity of SWNTs cause that they can form a three-dimensional conducting matrix which can be used for the immobilization of peroxidases. It was shown that the horseradish peroxidase adsorbed onto single-walled carbon nanotubes using the sodium cholate suspension-dialysis method was highly dispersed and retained a significant amount of the native peroxidase activity. In this biosensor L-cysteine residues were covalently bound to a gold electrode. Such a modified electrode showed high sensitivity towards hydrogen peroxide. The linear response of this biosensor was detected in the range of 1.0 × 10^−12^–1.0 × 10^−11^ M with the detection limit of 2.1 × 10^−13^ M mainly due to biocatalytic reduction of H_2_O_2_ based on direct electron transfer between the gold electrode and the active site of the peroxidase [78]. In turn, the horseradish peroxide biosensor with Au nanoparticle-dotted titanate nanotubes exhibited lower sensitivity towards H_2_O_2_. A linear response of this biosensor was obtained in the range of 15 × 10^−6^–750 × 10^−6^ M with the detection limit of 2.2 × 10^−6^ M. However, it exhibited stability with 90% of the detection signal retained over a two-week duration. In this biosensor titanate nanotubes play the role of the rigid material, whereas the hydrophobic ionic liquid was the entrapping reagent. The Au nanoparticle-dotted titanate nanotubes, exhibited a large surface area, good biocompatibility and provided a microenvironment which improves conductivity for the redox activity of peroxidase [72]. The same properties were observed after using nanocomposite film in the horseradish peroxidase biosensor based on alumina nanoparticles-chitosan. This biosensor, used for the determination of phenolic compounds, exhibited a linear response in the range of 5 × 10^−9^–7 × 10^−5^ M with the detection limit of 1 nM of hydroquinone and retained 80% of its initial activity to the reduction of hydroquinone after a month [73].

## 4. Immobilized Aromatic Ring Cleavage Dioxygenases in Bioremediation

Dihydroxylated aromatic compounds or their derivatives are substrates for the ring-cleaving dioxygenases. These enzymes couple O-O bond cleavage with the ring fission of hydroxylated derivatives either between two hydroxyl groups (*ortho* cleavage) or beside one of these (*meta* cleavage) [16,79]. 

**Figure 4:**
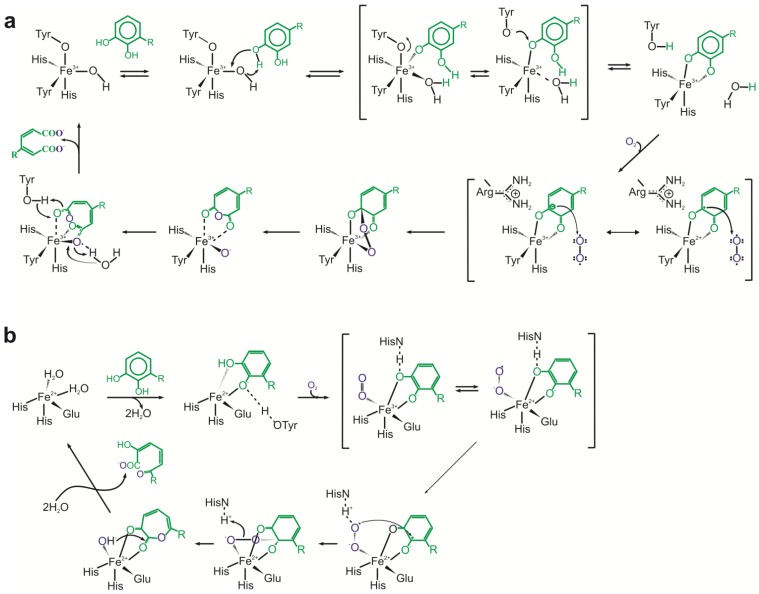
(**a**) Catalytic cycles of intradiol dioxygenases. (**b**) Catalytic cycles of extradiol dioxygenases [79,81,82,83].

Intradiol dioxygenases [E.C.1.13.11.X] catalyze cleavage of the aromatic ring at 1,2-position. The catalytic cycle involves binding of the substrate as a dianion and dioxygen to the metal in the active site of the enzymes. It forms a peroxide bridge between the ferric ion and substrate. In the next step, superoxide and substrate radicals recombine forming peroxo species. As a result, peroxide ligand undergoes the Criegee rearrangement with O-O bond cleavage and acyl group migration. As a consequence, anhydride is formed. Its hydrolysis leads to the formation of a final acyclic product (Figure 4a) [79,80,81].

Extradiol dioxygenases [E.C.1.13.11.X] catalyze the ring fission between hydroxylated and non-hydroxylated carbon of the aromatic ring. The catalytic mechanism of these enzymes starts with bidentate binding of the substrate as monoanion to the active-site iron (II) with simultaneous displacing of two water molecules. The binding of oxygen to the ferrous ion of the enzyme results in semiquinone-ferrous-superoxide intermediate formation. Subsequent Criegee rearrangement and O-O bond cleavage leads to lactone intermediate and ferrous-bound hydroxide ion creation. Hydrolysis of the lactone and release of the reaction product – semialdehyde – complete the reaction cycle (Figure 4b) [82,83].

Although dioxygenases have a significant potential in bioremediation of contaminated sites, their application in bioremediation processes is limited due to the low stability of the free enzyme and its propensity for substrates and/or product inhibition [18]. The presence of the ferrous ion at the catalytic centre of extradiol dioxygenases makes these enzymes very sensitive to oxidation and, consequently, to deactivation [17,18,84,85]. The protective effects of immobilization were observed for various catechol 2,3-dioxygenases after using of cyanogen bromide-activated agarose, alginate or κ-carrageenan as a carrier [17,18,85]. For example, catechol 2,3-dioxygenase from *Stenotrophomonas maltophilia* KB2 immobilized in calcium alginate gel exhibited almost 51% of its initial activity after 35 days of incubation at 4 °C, whereas the free enzyme was inactive after 24 h of storage [18]. The inactivation of catechol 2,3-dioxygenases is also frequently observed in the presence of compounds such as sodium azide, aliphatic alcohols or phenols, which may coordinate ferrous ion [17,18,86]. 

Wojcieszyńska *et al.* [18] showed that sodium azide did not affect the activity of the immobilized catechol 2,3-dioxygenase while it inhibited the free enzyme at all tested concentrations (0.1–0.3 mM). The protective effect of immobilization depends on the kind of carrier used. For example, κ-carrageenan as a matrix, did not prevent ferrous ion in the catalytic center of catechol 2,3-dioxygenase [17], while the immobilization of catechol 1,2-dioxygenase in calcium alginate gel improves activity of the enzyme in the presence of an organic solvent. Probably, a highly hydrophilic environment surrounding the enzyme immobilized in alginate gel protects it against the organic solvent [87,88].

One of the enzyme’s features desirable for its application in bioremediation processes is broad substrate specificity. It is generally known that the immobilization of enzymes may cause conformational changes of protein structure. These changes may cover the hydrophobic channel in the active site of the enzyme by which the substrate penetrates into the active site. This in turn results in the limitation of local concentration of the substrate and, consequently increases the resistance of the enzyme to substrate toxicity. This effect probably caused wider substrate specificity of catechol 2,3-dioxygenase from *Stenotrophomonas maltophilia* KB2 after its immobilization in calcium alginate and κ-carrageenan beads [17,18]. Similar results were observed by Iwaki and Nozaki [85] after using pyrogallol, protocatechuic acid and 3,5-dichlorocatechol as a substrate for metapyrocatechase immobilized on cyanogen bromide-activated agarose. Entrapment of intradiol dioxygenase, protocatechuate 3,4-dioxygenase from KB2 strain in calcium alginate hydrogel or glyoxyl agarose increased activity of the enzyme towards different aromatic acids. However, activity of protocatechuate 3,4-dioxygenase toward examined substrates depended on a type of carrier used during immobilization. Significantly higher activity of the enzyme was observed after its immobilization in calcium alginate gel than on glyoxyl agarose. It was probably connected with strong electrostatic interaction between positively charged amino acid residues of the enzyme and negatively charged groups of sodium alginate [16]. 

Since the bioremediation processes depend on various environmental factors, the effective application of enzymes in these processes depends on their resistance to changing conditions. The most important factors that affect the enzymes’ activity are pH and temperature [84]. Although after immobilization significant changes in the enzymes’ pH profile were not observed, it may change their temperature optimum [17,18,85,88,89,90]. Silva *et al.* [89] demonstrated that the immobilization of catechol 2,3-dioxygenase from *Gordonia polyisoprenivorans* on sodium alginate matrix increased temperature range of the enzyme. A similar effect was observed for hydroxyquinol 1,2-dioxygenase after its immobilization onto single-walled carbon nanotubes [90]. In turn, after the immobilization of 1,2-dioxygenases from *Acinetobacter radioresistens* S13, *Pseudomonas putida* and *Mycobacterium fortuitum* on nanosponges or sodium alginate gel the optimum temperature for examined enzymes was shifted toward higher values [88,91,92]. Similar results were obtained after the immobilization of catechol 2,3-dioxygenase from *Bacillus stearothermophilus* and *Stenotrophomonas maltophilia* KB2 on activated glyoxyl agarose and sodium alginate beads, respectively [18,84]. The replacement of alginate to κ-carrageenan during the immobilization of catechol 2,3-dioxygenase from KB2 strain shifted the optimum temperature towards lower values [17]. The immobilization of protocatechuate 3,4-dioxygenase from this strain in calcium alginate or glyoxyl agarose resulted in the decrease of the optimum temperature by 5 °C and 10 °C, respectively [16]. Moreover, the formation of covalent bonds between the enzyme and glyoxyl agarose caused the increase of enzyme rigidity and, in consequence, the activity of the immobilized enzyme was higher than the free enzyme [16]. These results indicate the possibility of controlling dioxygenases activity depending on the immobilization method [17].

## 5. Conclusions

The development of immobilization methods caused a significant increase in the application of oxidoreductases in various technological processes. The use of different kinds of carriers enables to reuse and recover enzymes which resulted in a significant reduction in bioprocesses costs. Moreover, as a result of the protective effect of the matrix, immobilized enzymes become more resistant to changes of environmental parameters such as temperature, pH or inhibitory effect of different compounds. This in turn improves enzyme operational stability. 

Nevertheless, the practical applications of immobilized enzymes in biotechnological processes on a large scale are still limited. Endless search for new supports for immobilization is not possible. It is also known that protein engineering or immobilization individually cannot make an ideal catalyst for industrial processes. However, improvement of the properties of the immobilized biocatalyst may be achieved through the combination of enzyme immobilization with different tools, which enable genetic engineering or chemical modification of both enzymes and support. Chemical modification of the support may change the nature of the hydrophobic matrix by incorporation of different groups such as epoxy groups. The usage of modified support improves the immobilization rate and orientation of the protein on the support. Likewise genetic engineering and/or chemical modification of proteins allow immobilization of enzymes at a well-defined position. Exchange of the amino acids present on the protein surface on the specific group such as thiol groups, makes possible the creation of affinity bonds between activated matrix and enzyme [93,94,95,96,97,98,99]. It seems that broadening of enzymes’ applications in biotechnological processes will be possible thanks to the site-specific immobilization of enzymes, which is a very good basis for the optimization of reaction conditions [93,94,95,96,97,98,99]. 
